# Factors that affect placental retinol transfer in preterm infants and mothers with retinol deficiency

**DOI:** 10.3906/sag-2102-301

**Published:** 2021-07-10

**Authors:** Kadir Şerafettin TEKGÜNDÜZ, Dilara DILEK, Mustafa KARA, Nurinnisa ÖZTÜRK, Sibel Ejder TEKGÜNDÜZ

**Affiliations:** 1 Department of Pediatrics, Division of Neonatology, Faculty of Medicine, Atatürk University, Erzurum, Turkey; 2 Department of Pediatrics, Faculty of Medicine, Atatürk University, Erzurum, Turkey; 3 Department of Pediatrics, Division of Neonatology, Faculty of Medicine, Atatürk University, Erzurum, Turkey; 4 Department of Medical Biochemistry, Faculty of Medicine Atatürk University, Erzurum, Turkey; 5 Department of Gynecology and Obstetrics, Erzurum Regional Training and Research Hospital, Erzurum, Turkey

**Keywords:** Preterm infant, retinol, placenta

## Abstract

**Background/aim:**

The retinol level and retinol delivery to the placenta may vary depending on various factors involving the mother and newborn. The present study evaluates the factors affecting retinol levels in newborns and the transplacental retinol passage in preterm newborns.

**Materials and methods:**

In this prospective cohort study, the retinol and retinol binding protein (RBP) in the umbilical cord blood of 44 preterm infants with a gestation age of <30 weeks were studied. Serum retinol and RBP levels were determined using an enzyme-linked immunosorbent assay, and the rate of transplacental retinol passage was calculated. The demographic data of mothers and newborns, the use of vitamins by the mother, the application of antenatal corticosteroids, and any diseases diagnosed during pregnancy were recorded. An evaluation was made of the retinol, RBP, and other factors of the mother and newborn affecting transplacental retinol passage.

**Results:**

A retinol deficiency was identified in 68.2% of the study population. Retinol and RBP levels in umbilical cord blood (273.7 ± 150.03 ng/mL, 7.88 ± 5.6 ng/mL, respectively) were significantly higher than the corresponding levels in the mother (206.4 ± 86.26 ng/mL, 1.04 ± 0.97 ng/mL, respectively). Umbilical cord blood retinol deficiency was more common in the male participants, while the transplacental retinol passage rate was higher in females. The umbilical cord blood RBP was found to be lower in those administered antenatal corticosteroids than in those who did not receive antenatal corticosteroids, and median maternal RBP levels were lower in patients with anemia and pregnancy-induced hypertension than in those with no disease.

**Conclusion:**

Placental adaptation and contributing factors may vary in populations with severe retinol deficiency. The finding of significantly increased cord blood retinol levels when compared to maternal retinol levels in the present study suggests that some compensatory mechanisms, such as increased placental RBP levels, support the presentation of retinol to the fetus, even if the mother has a retinol deficiency.

## 1. Introduction

Retinol is an essential vitamin that plays a role in the expression of many genes and in the growth of the fetus. Retinol plays a critical role in pregnancy with regard to rapid cell division and growth [1,2]. Retinol is necessary for the development of multi-organ systems, including the immune system, lungs, eyes and thyroid [2,3]. According to the World Health Organization (WHO) data, retinol (vitamin A) deficiency (<0.7 mmol/L or <20 μg/dL) is a significant public health problem with a considerable contribution to mortality, especially in developing countries (4). Malnourishment in pregnant women has been reported to cause chronically low levels of maternal retinol stores in countries with a low income levels [4]. Fetal retinol deficiencies can lead to many congenital abnormalities, with links identified between embryonic retinol deficiency and cleft lip/cleft palate, anophthalmia or microphthalmia, urogenital system abnormalities, abnormalities of the heart and large vessels, and extremity malformations [5–7].

Retinol synthesizes de novo [8], starting in the early intrauterine period, when the fetus is dependent on the mother’s retinol in whom it is stored in the liver and released into the blood circulation together with the retinol-binding protein (RBP), as a transport protein [8,9]. RBP is a protein with a 21 kDa weight that binds a single retinol molecule, and that is involved in the transport of retinol to peripheral tissues or the embryo [9–11]. The retinol-RBP complex comprises 95%–99% of all retinoids in circulation, and is present at a 1:1 molar rate [8,12]. The retinol intake of the fetus is maintained at a stable level through possible maternal hemostatic mechanisms, even though fluctuations may occur in maternal retinol levels [13]. The maternal retinol-RBP complex is separated in the blood-placental barrier, creating free retinol that passes through the placenta in maternity [10]. Maternal RBP has been shown not to pass the placenta [8,10]. Fetal RBP is produced in the yolk sac endoderm and in the placental uterine intersection, starting in the early intrauterine period [14], although fetal RBP does not pass into the maternal circulation [10].

Preterm newborns are known to have lower serum and hepatic retinol levels than term newborns [12,15], although it has been reported that retinol deficiency is more common in the mothers of newborns with low serum retinol levels at birth [16]. Newborns have lower capacity for hepatic retinol storage due to the low maternal retinol levels during pregnancy and the selective blood-placental barrier, and this also protects the fetus from the possible teratogenic effect of retinol [17]. That said, data is limited on the role of the placenta in the retinol intake of mothers with retinol deficiency. Normal development in the embryo has been demonstrated in animal models in the absence of maternal RBP and in cases of sufficient retinol intake [10], and in such cases, even though hepatic storage is inadequate, the retinol intake in the diet has been reported to be adequate for the development of the fetus [10]. Placental retinol passage is known to increase in cases of maternal retinol deficiency [18]. Placental regulatory mechanisms might be effective in the transfer of retinol, which is known to have a teratogenic effect when deficient or present in excessive amounts [10,18,19]. In animal models, placental RBP has been shown to play a key role in fetal retinol utility in cases of maternal retinol deficiency [10].

This is the first study in Turkey to evaluate the factors affecting maternal and umbilical cord blood retinol and RBP levels, as well as the rate of transplacental retinol passage in preterm newborns. Determining the retinol levels and the associated factors in the pregnant population in every country, and even in every region, is important for public health.

The present study evaluates the factors affecting umbilical cord blood and maternal retinol and RBP levels, as well as transplacental retinol passage in preterm newborns.

## 2. Materials and methods

This cohort study was conducted prospectively between June 2020 and January 2021 in the Obstetrics and Gynecology Clinics of Atatürk University. Ethics Board approval was obtained from the Ethics Board of the Medical School of Atatürk University (Decision no: 2020-7/29). Signed informed consent was obtained from the families prior to patient selection.

### 2.1. Study population

Included in the study were preterms born in our hospital at gestational week <30, while mothers or newborns with a major abnormality or known congenital metabolic disease, mothers with a chronic liver, kidney or gastrointestinal system disease, and those who did not give consent were excluded.

The sex and birth weight of the newborns, and the gestation week, maternal age, gravidity, delivery mode, vitamin intake, vitamin A use during pregnancy, and antenatal steroid treatment in the mothers were recorded. Also recorded were any preterm premature ruptures of membranes (PPROM) diagnosed during pregnancy, as well as preeclampsia/eclampsia, pregnancy-induced hypertension, urinary infection, gestational diabetes mellitus, and anemia, if present.

PROM refers to the rupture of membranes prior to the onset of labor, while membrane ruptures before labor and before 37 weeks of gestation is referred to as preterm PROM [20].

Preeclampsia/eclampsia, and pregnancy-induced hypertension were defined according to the diagnostic criteria specified in the current American College of Obstetricians and Gynecologists (ACOG) guidelines [21].

Those with at least one urine culture yielding a bacterial growth of 10^5^ colony-forming units/mL of urine during pregnancy were recorded as having a urinary infection [22].

Gestational diabetes mellitus is defined as glucose intolerance diagnosed for the first time during pregnancy based on fasting blood glucose or glucose tolerance tests [23].

Anemia in pregnancy is defined as Hb <11 g/dL in the first trimester, <10.5 g/dL in the second trimester and <11 g/dL in the third trimester [24].

### 2.2. Sampling and test study method

Approximately 6 ml of umbilical cord blood was drawn at birth from all newborns included in the study by experienced individuals using a vacutainer, and the same amount of venous blood was drawn into a biochemistry tube from the mothers’ antecubital region immediately after birth, and centrifuged for 10 min at 4500 rpm after the completion of coagulation, and the sera were separated. The serum samples were frozen and stored at −80 ° until the time of analysis, and were thawed in appropriate ways, with all analyses carried out in the Medical Biochemistry Laboratory of Atatürk University at a single session.

The retinol levels of the serum samples were analyzed using ELISA kits obtained from the Bioassay Technology Laboratory (E1548 Hu Jiaxing, Zhejiang, China), RBP levels were analyzed with ELISA kits provided by Elabscience (E-EL-H1581 Texas, USA) following the standard manufacturers protocol and using a Dynex automated ELISA reading device (Dynex Technologies Headquarters, Chantilly, USA). The measurement ranges of the kits for retinol and RBP were 2–800 ng/mL and 0.07–100 ng/mL, respectively. Both intra-coefficient variability and inter-coefficient variability are < 10%.

The formula “umbilical cord blood retinol/maternal retinol ×100” was used to calculate the placental retinol passage rate, identifying the percentage of maternal retinol passing into the umbilical cord blood [18].

Retinol deficiency was evaluated based on WHO guidelines [4], with a level of <20 μg/dL accepted as a deficiency. A deficiency level of <200 ng/mL was accepted to match the ng/mL unit adopted in the present study.

### 2.3. Statistical analysis

The statistical analysis was carried out using IBM SPSS Statistics v. 23.0 (IBM Corp., Armonk, NY, USA). Descriptive statistics were applied for distributions (number and percentage), and numerical properties for numerical data (mean, median, std. deviation, minimum and maximum). Shapiro–Wilk and Kolmogorov–Smirnov tests were used to analyze the normality of the distribution. G-power software (v. 3.1.9.2, Kiel, Germany) was used to determine the sample size. The number of patients required was determined 42 for t-test analysis of newborns and mother’s groups with 0.55 effect size, 5% type I error, and 80% power. Parametric tests were used to compare maternal retinol and RBP, and umbilical cord blood retinol and RBP levels and the groups with normal distribution of these levels (sex, gravidity, antenatal corticosteroids). Nonparametric tests were used to compare retinol and RBP levels in maternal disease groups. A t-test was used for the comparison of parametric tests, the Mann–Whitney U test for the comparison of two groups, and the Kruskall–Wallis test for the comparison of nonparametric data in more than two groups. Pair-wise comparisons were performed using Dunn’s post-hoc test. A Pearson correlation test was used to evaluate whether the continuous variables were correlated. A Chi-square test was used for the comparison of categorical variables with each other. A two-tailed p-value of <0.05 was accepted as significant.

## 3. Results

During the study period, 76 newborns under 30 weeks’ gestation were born at our hospital, and 44 of these premature newborns who met the inclusion criteria and whose family provided consent were included in the study. Of these, 22 (50%) preterms were female and 22 (50%) were male. The median age of gestation and birth weight were 27.8 ± 2 weeks and 1007 ± 300 g, respectively. The maternal mean age was 26.9 ± 6.5 years and the mothers’ pregnancies were numbered between 1 and 9. Among the mothers, 16 were primiparous (36%) and 28 were multiparous (64%). There was no multiple pregnancy (twins or more) in our study. Three (6.8%) newborns were delivered vaginally. All 41 (93.2%) mothers who had cesarean delivery were administered general anesthesia. Only 3 (6.8%) mothers used vitamins (folic acid and/or multivitamins) for 30–45 days during pregnancy. None of the mothers received additional vitamin A supplements. Among the mothers, 26 were administered antenatal corticosteroids (59%) while 18 were not (41%).

The mean maternal retinol and RBP level was 206.4 ± 86.2 ng/mL and 1.04 ± 0.97 ng/mL, respectively, and the mean umbilical cord blood retinol and RBP levels were 273.7 ± 150 ng/mL and 7.88 ± 5.6 ng/mL, respectively. Umbilical cord blood retinol levels and RBP were statistically significantly higher than the respective maternal levels (p: <0.001 and p: 0.007, respectively) (Table 1). A positive correlation was identified between the maternal and umbilical cord blood retinol levels (r: 0.42, p: 0.04), while no correlation was found between the umbilical cord blood RBP and the maternal RBP levels (r: 0.07, p: 0.65). The rate of retinol deficiency (<200 ng/mL) was found to be higher in the maternal blood (30, 68.2%) than in the umbilical cord blood (18, 40.9%) (p < 0.001).

No associations were identified between the maternal and umbilical cord blood retinol and RBP levels, nor the birth weight, gestational week or maternal age (Table 2). No effects of gestational week, birth weight and maternal age were identified in the presence of retinol deficiency in the umbilical cord blood or in maternal samples (Table 3), and no significant difference was found between sex and the maternal and umbilical cord blood retinol RBP levels (Table 4). However, when the distribution of retinol deficiency was evaluated, umbilical cord blood retinol deficiencies were identified in 12 of the 22 (54.5%) male newborns and five of the 22 (22.7%) female newborns. Umbilical cord blood retinol deficiencies were found to be significantly more common among the male newborns than the female newborns (p: 0.03) (Table 3).

In this study group, the umbilical cord blood and maternal retinol levels in mothers who had received vitamins were similar to those of the mothers who received no vitamin supplements (p:0.23 and p:0.22 respectively). No statistical analysis was made of this group to evaluate any deficiencies, since vitamin use was limited and the duration of vitamin intake was short (30–45 days). Statistical analysis could also not be made of the mode of delivery and the method of anesthesia, since there were only three patients delivered vaginally and all mothers undergoing cesarean section were administered general anesthesia.

An analysis of the mothers who had been administered antenatal corticosteroids and those who were not revealed mean umbilical cord blood RBP levels of 5.9 ± 3.07 ng/mL and 10.6 ± 7.3 ng/mL, respectively, being was significantly lower in the group administered corticosteroids (p:0.006) (Table 4). No difference was found in the frequency of maternal and umbilical cord blood retinol deficiencies among those with and without antenatal steroid application (Table 3).

No accompanying disorder was identified in 20 of the mothers (45%), while preeclampsia was present in 9 (20%), PPROM in 4 (9%), pregnancy-induced hypertension in 6 (13.6%), urinary infection in 3 (6.8%), and anemia in 2 (4.5%). When the comparison between the umbilical cord blood retinol-RBP and maternal retinol-RBP levels in those diseases was evaluated, maternal RBP levels were found to differ in different maternal diseases, to a statistically significant degree (p:0.02) (Table 4). In a subgroup analysis, the median maternal RBPs were found to be 0.99 ng/mL (0.05–24.9 ng/mL), 0.51 ng/mL (0.21–1.30 ng/mL) and 0.11 ng/mL (0.02–0.21 ng/mL) in mothers with no accompanying disease, with pregnancy-induced hypertension and with anemia, respectively. Maternal RBP levels were found to be lower in the hypertension and anemia groups than in mothers with no additional disease (p: 0.04 and p: 0.01 respectively) (Table 4). In terms of the frequency of maternal and umbilical cord blood retinol deficiency, the two groups with and without additional diseases were compared, revealing no significant differences (Table 3).

The mean rate of transplacental retinol passage was 134.1 ± 43.5%. A negative correlation was identified between maternal RBP levels and transplacental passage rate (r: −0.38, p: 0.01). In contrast, no correlation was found between umbilical cord blood RBP levels and the transplacental passage rate (r: 0.25, p: 0.09), although the mean transplacental passage rate was 148.4 ± 46.8% and 119.7 ± 35.4 % in the female and male newborns, respectively, being significantly higher in the female group (p:0.02). (Table 5).

## 4. Discussion

The detection of retinol deficiency in more than 20% of a society is a serious public health problem, according to WHO criteria [4]. In the present study group, retinol deficiencies were present in 68.2% of mothers and in 40.9% of preterm infants, indicating a severe retinol deficiency in the study group. The maternal/fetal retinol concentration is around 2:1 in mothers in the absence of severe retinol deficiency [15]. Low retinol levels in the newborns of mothers with retinol deficiency have been reported, and there are also reports of transplacental retinol passage being increased in this group [18,25]. A positive correlation was found in the present study between maternal and cord blood retinol levels. It is worthy of note, however, that umbilical cord blood retinol levels were statistically significantly higher than maternal retinol levels. The vitamin A metabolism and its placental organization is a complex process, and there are some as yet unknown mechanisms [13,15]. These results suggest that fetus-preserving placental homeostatic mechanisms are effective even in the case of severe retinol deficiency in mothers, and that the recommended daily vitamin A intake in diet may be sufficient, as mentioned in the animal model study by Quadro et al. [10]. Nevertheless, in literature, fetal vitamin A levels are reported to be relatively stable, despite fluctuations in maternal retinol levels [26]. None of the patients in the present study had teratogenicity related to retinol deficiency.

In the present study, cord blood RBP levels were also markedly higher than the maternal RBP levels, indicating increased placental RBP in mothers with retinol deficiency. Placental homeostasis has plays a key role in the delivery of retinol to the fetus, and is responsible for the storage of retinoids until the fetal liver maturation is complete [27,28]. It has been further reported that the homeostatic mechanisms that affect maternal RBP levels are also effective in retinol delivery to the fetus [26]. In the present study, a negative correlation was identified between maternal RBP levels and transplacental retinol transfer. It is possible that maternal RBP levels, and in turn, placental retinol delivery, is decreased due to the increased passage of transplacental retinol to protect the fetus from a sudden increase in retinol levels.

Serum retinol levels are lower in preterm newborns than in full-term newborns [29–32], although contradictory findings have been reported regarding the effects of gestational week on serum retinol levels in premature newborns [16,33–35]. In their autopsy study, Shenai et al. evaluated 25 newborns with extremely low birth weight (ELBW) and reported no difference in serum and hepatic vitamin A levels, based on sex, race, gestational week and birth weight [33]. Tao et al. reported no association between <35 weeks’ gestational age and < 2500 g birth weight with umbilical cord blood vitamin A deficiency (< 0.7 mmol/L) [34]. Conversely, there have been studies reporting lower serum vitamin A levels in the male sex than in the female sex in literature [36–38]. In the present study, no association was found between the maternal and umbilical cord blood retinol levels and sex, birth weight, gestational week, maternal age or gravidity, while the frequency of umbilical cord blood retinol deficiency was significantly higher in male newborns when compared to female newborns. We also found transplacental retinol passage to be higher in the female sex when compared to the male sex. The lesser frequency of umbilical cord blood retinol deficiency in females might be attributed to this finding. Nevertheless, there is a lack of adequate data explaining how the placental vitamin A metabolism changes based on the fetal sex.

Different results have been reported on the effect of antenatal corticosteroids on umbilical cord blood retinol levels [39,40]. Inder et al., in their study evaluating the association of retinol levels in the newborn and respiratory outcome in 57 newborns with extremely low birth weights, reported that antenatal steroid application increased the umbilical cord blood retinol level [39]. Chen et al. reported that antenatal steroid applications had no effect on the frequency of serum retinol deficiency (<20 μg/dL) in samples taken within the first 48 h of birth in their study of 115 premature newborns at <29 weeks of gestation and/or <1250 g birth weight [40]. In the present study, umbilical cord blood retinol, and maternal retinol and RBP levels were found to be higher in the group treated with antenatal corticosteroids than in those who were not, although not significantly. In addition, it was determined that antenatal corticosteroids had no effect on the transplacental passage rate of retinol. In contrast, umbilical cord blood RBP levels in the present study was statistically significantly lower in the group administered antenatal corticosteroids than in those not treated with antenatal corticosteroids. It has been reported in literature that maternal and umbilical cord blood RBP levels increase with the administration of antenatal corticosteroids [41,42], although simultaneous retinol levels were not investigated in those studies, and so no data was provided on how antenatal corticosteroids could affect umbilical cord blood RBP levels in the presence of retinol deficiency [41,42]. Georgieff et al. reported the potential of RBP synthesis to be increased in newborns due to the additive effect of antenatal corticosteroids on hepatic maturation [42]. The role of placental RBP in the retinol metabolism of preterm newborns has been well documented [10,14]. We suggest that antenatal corticosteroids could affect placental homeostasis differently in situations in which maternal retinol deficiency is more frequent, as seen especially in our study population.

Retinol deficiency in the mother may have some negative consequences during pregnancy. In Cochrane’s meta-analysis, it was reported that vitamin A intake, along with iron and folic acid supplementation, may decrease the incidence of anemia in pregnancy in mothers with vitamin A deficiency [43]. Radhika et al. reported vitamin A deficiency in mothers to increase the rate of anemia [44], while there have been reports on preeclampsia stating that maternal RBP levels have decreased, increased or remained unchanged [45–47]. It has been further reported that maternal RBP associated with gestational diabetes and increased RBP is a marker of insulin resistance [48]. Inoue et al. found that maternal RBP levels increased and umbilical cord blood RBP levels decreased in the presence of pregnancy-induced hypertension, and it has been reported that this condition may be attributable to insulin resistance in the mother [49]. No association has been found between preeclampsia, urinary infection and PPROM, and maternal-umbilical cord blood retinol and RBP levels. Maternal RBP levels were found to be lower in patients with pregnancy-induced hypertension and anemia than in those with no additional disease. Hypertension and anemia can have multiple causes, such as malnutrition during pregnancy [50,51]. In the present study, the significantly low levels of retinol in this patient population indirectly suggested malnutrition in the study population, and the low levels of maternal RBP in the study among patients with hypertension and anemia may also be due to malnutrition.

This study has some limitations. The high rate of retinol deficiency in our region was an unforeseen condition, and therefore, risk factors such as maternal nutrition were not considered as a cause of retinol deficiency. In addition, due to the COVID-19 pandemic, the number of patients recruited was lower than expected, the study having been planned prior to the pandemic. The numbers were small, particularly in some patient groups, in order to evaluate the effects of maternal diseases on retinol metabolism. Besides, the effect of the mode of delivery and the method of anesthesia could not be evaluated in those who had cesarean delivery Finally, the effect of placental insufficiency on retinol metabolism was not studied, as placental insufficiency was not evaluated.

In conclusion, placental retinol passage is regulated according to the presence of a maternal deficiency of retinol. Retinol is essential for the fetus, while excessive amounts are undesirable, and different factors affect its levels in various populations. Significantly high umbilical cord blood retinol and RBP levels when compared to maternal retinol and RBP levels, as was the case in some cases in the present study, suggest that some of the known factors that affect the transplacental retinol passage could be adjusted in populations with maternal retinol deficiencies. That said, studies of the placental compensation in cases of retinol deficiency involving larger populations are required.

## Figures and Tables

**Table 1:** Comparison of retinol and RBP values studied in maternal and cord blood.

	Mean (ng/mL)	P
**Cord blood retinol** ≠	273.7 ± 150	**<0.001**
**Maternal retinol** ≠	206.4 ± 86.2≠	
Cord blood RBP*,≠	7.88 ± 5.6	**0.007**
Maternal RBP*,≠	1.04 ± 0.97	

* RBP: Retinol binding protein.

≠ mean ± standard deviation.

**Table 2:** Correlations between retinol and RBP levels and demographic data.

	UCB* retinol		Maternal retinol		UCB* RBP***		Maternal RBP***	
	R **	P	R **	P	R **	P	R **	P
**Gestational age**	− 0.09	0.52	− 0.01	0.92	0.03	0.81	0.01	0.91
**Birth weight**	− 0.06	0.67	0.08	0.57	− 0.15	0.37	0.11	0.45
**Maternal age**	− 0.15	0.32	− 0.12	0.42	0.04	0.79	0.2	0.19

* UCB: Umbilical cord blood.

** R: Correlation coefficient.

*** RBP: Retinol binding protein.

**Table 3:** Comparison of groups according to the umbilical cord blood and maternal retinol deficiency.

		UCB* retinol deficiency (n = 18)	UCB* retinol-normal (n = 26)	P*	Maternal retinol deficiency (n = 30)	Maternal retinol-normal (n = 14)	P*
**Gestational age (week)** ≠		28.3 ± 1.7	27.5 ± 2.1	0.16	27.9 ± 2.1	27.6 ± 1.7	0.63
**Birth weight (g)** ≠		1050 ± 262	977 ± 326	0.43	1002 ± 328	1015 ± 241	0.89
**Maternal age (year)** ≠		29.3 ± 7	27.5 ± 5.7	0.06	26.9 ± 7	26.8 ± 5.6	0.97
**Sex** €	Female (n = 22)	5 (22.7%)	17 (77.3%)	**0.03**	16 (72.7%)	6 (27.3 %)	0.74
	Male (n = 22)	13 (54.5%)	9 (45.5%)		14 (63.6%)	8 (36.4 %)	
**Gravidity** €	Primiparous (n = 16)	4 (25%)	12 (75 %)	0.12	10 (62.5)	6 (37.5 %)	0.73
	Multiparous (n = 28)	14 (50%)	14 (50 %)		20 (71.4%)	8 (28.6 %)	
**Antenatal corticosteroids** €	None (n = 18)	8 (44.4%)	10 (55.6 %)	0.76	13 (72.2%)	5 (27.8 %)	0.74
	Present (n = 26)	10 (38.4%)	16 (61.6 %)		17 (65.3%)	9 (34.7 %)	
**Maternal disease** €	None (n = 20)	7 (35%)	13 (65 %)	0.54	12 (60%)	8 (40 %)	0.34
	Present (n = 24)	11 (45.8%)	13 (54.2 %)		18 (75%)	6 (25 %)	

* UCB: Umbilical cord blood.

≠ mean ± standard deviation.

€ number (%).

**Table 4:** Comparison of mean maternal and umbilical cord blood retinol and mean RBP with sex, gravidity, antenatal corticosteroids use, and maternal diseases.

		UCB* retinol (ng/mL)	P	Maternal retinol (ng/mL)	P	UCB* RBP** (ng/mL)	P	Maternal RBP** (ng/mL)	P
**Sex** ≠	Female	303.1 ± 184	0.2	203.1 ± 93.76	0.79	9.1 ± 7.2	0.14	0.81 ± 0.54	0.11
	Male	244.3 ± 102.1		209.8 ± 80.1		6.6 ± 3.1		1.29 ± 1.24	
**Gravidity** ≠	Primiparous	272.5 ± 151.6	0.96	196.8 ± 52.6	0.58	8.35 ± 3.5	0.68	1.17 ± 1.15	0.55
	Multiparous	274.4 ± 152.1		212 ± 101		7.6 ± 6.6		0.98 ± 0.86	
**Corticosteroids** ≠	None	260.1 ± 145.6	0.62	190.3 ± 56.4	0.3	10.6 ± 7.3	**0.006**	1.13 ± 1.09	0.79
	Present	283.1 ± 155.1		217.6 ± 101.5		5.9 ± 3		1.01 0.86	
**Maternal disease** α	None	209 (147–746)	0.55	182 (124–441)	0.71	6.8(0.87–13.8)	0.1	0.99 (0.05–	**0.02**
	Preeclampsia	195 (176–680)		182 (137–297)		8.2 (2.6–6.3)		0.52 (0.21–3.44)	
	PPROMβ	265 (190–421)		207 (144–249)		6.5 (1.5–8.3)		0.56 (0.16–1.21)	
	Hypertension	206 (183–663)		159 (123–496)		6.2 (0.7–8.3)		0.51 (0.21–1.3)	
	Urinary infection	191 (187–231)		175 (124–181)		12.7 (8.7–37.8)		1.32 (1.07–4.2)	
	Anemia	439 (261–617)		180 (141–218)		10.8 (9.2–12.4)		0.11 (0.02–0.21)	

* UCB: Umbilical cord blood.

** RBP: Retinol binding protein.

≠ mean ± standard deviation.

α median (range).

β PPROM, preterm premature rupture of membranes.

**Table 5:** Comparison of transplacental retinol passage.

		Transplacental retinol passage (%)	P
**Sex** ≠	Female	148.4 ± 46.8	**0.02**
	Male	119.7 ± 35.4	
**Gravidity** ≠	Primiparous	139 ± 58.3	0.5
	Multiparous	130 ± 33	
**Antenatal corticosteroids** ≠	None	138.2 ± 54.6	0.6
	Present	131.2 ± 34.7	
**Maternal disease** ≠	None	121.9 ± 41.1	0.09
	Present	144.2 ± 43.7	

≠ mean ± standard deviation.
